# Assessment of antimicrobial mismatches in empirical treatment in early PJI after aseptic revision arthroplasty

**DOI:** 10.1093/jacamr/dlac124

**Published:** 2022-12-07

**Authors:** C M Veerman, J H M Goosen, D S C Telgt, W H M Rijnen, M H Nabuurs, H F L Wertheim

**Affiliations:** Department of Orthopedics, Sint Maartenskliniek, Nijmegen, The Netherlands; Department of Internal Medicine, Radboud University Medical Centre, Nijmegen, The Netherlands; Department of Orthopedics, Sint Maartenskliniek, Nijmegen, The Netherlands; Department of Orthopedics, Sint Maartenskliniek, Nijmegen, The Netherlands; Department of Internal Medicine, Radboud University Medical Centre, Nijmegen, The Netherlands; Department of Orthopedics Nijmegen, Radboud University Medical Centre, Nijmegen, The Netherlands; Department of Medical Microbiology, Canisius Wilhelmina Ziekenhuis, Nijmegen, The Netherlands; Department of Medical Microbiology, Radboud University Medical Centre, Nijmegen, The Netherlands

## Abstract

**Background:**

In early periprosthetic joint infection (PJI), ‘debridement, antibiotics and implant retention’ (DAIR) is a widely accepted form of treatment. Empirical antimicrobial treatment is started while culture results of tissue samples taken during debridement are pending.

**Objectives:**

In this retrospective study we assessed the antimicrobial mismatch rate between empirical treatment and the susceptibility of the causative microorganisms of PJI after aseptic revision arthroplasty. We analysed risk factors for antimicrobial mismatches and the impact of mismatches on the outcome of PJI treatment.

**Results:**

A total of 119 patients were included in the analysis. In 72% (86/119) of the cases there was an antimicrobial mismatch in empirical treatment. Most of the antimicrobial mismatches were caused by multidrug-resistant (MDR) *Staphylococcus* spp. (77%, 66/86). In multivariable analysis, polymicrobial PJI was significantly associated with antimicrobial mismatch (OR: 6.89; 95% CI: 2.38–19.53; *P* < 0.001), and antimicrobial mismatch was significantly associated with reduced success rate of PJI treatment (OR: 0.20; 95% CI: 0.05 ± 0.82; *P = *0.026). There was no difference in successful outcome between PJI caused by Gram-negative bacilli (61%) and Gram-positive bacteria (69%, *P* = 0.516).

**Conclusions:**

Mismatching empirical antimicrobial treatment after DAIR following aseptic revision arthroplasty was significantly associated with failure of PJI treatment. Polymicrobial PJI is a risk factor for antimicrobial mismatch of the empirical treatment of PJI. Antimicrobial mismatch and delay in targeted treatment should be integrated in the approach to optimize antibiotic treatment to improve clinical outcomes, while minimizing unintended side effects of antimicrobial use (antimicrobial stewardship).

## Introduction

Periprosthetic joint infection (PJI) is a feared complication of joint replacement therapy. ‘Debridement, antibiotics and implant retention’ (DAIR) is an accepted form of treatment of early and haematogenous PJI.^1,2^ Empirical antimicrobial treatment is started awaiting the culture results of tissue samples taken during debridement. The choice for the empirical antimicrobial treatment depends on the local aetiology of PJI and the side effects of the treatment: the antimicrobial treatment should be broad enough to target the most frequently identified causative microorganisms, while avoiding potential toxicity and the development of antimicrobial resistance.^3^ Gram-positive bacteria, especially staphylococci, are the most common causatives of PJI.^4,5^ Based on this, cefazolin or vancomycin are agents of choice for empirical antimicrobial treatment depending on local resistance patterns. In our previous study analysing the outcome results of DAIR performed after revision arthroplasty for suspected early PJI, we found a high antimicrobial mismatch rate between empirical treatment and the causative microorganisms of PJI.^6^ The antimicrobial mismatch was associated with poor outcome results of DAIR after revision arthroplasty. This study was a single-centre study and both septic and aseptic revisions were included. As a result of the heterogeneous cohort there was a high variation in type of index revisions that were included and prior use of antimicrobial treatment, which might have influenced the risk of mismatching empirical antimicrobial treatment. Because of the heterogeneous cohort and the association of antimicrobial mismatch with reduced outcome of DAIR, we performed a multicentre follow-up study focusing on a more homogeneous cohort of patients with early PJI after aseptic revisions. We describe the aetiology of causative microorganisms of PJI, the antibiotic susceptibility of causatives and the empirical antimicrobial treatment that was used. We analysed risk factors for antimicrobial mismatching of the empirical treatment and related the antimicrobial mismatch to the outcome results of PJI treatment.

## Patients and methods

### Patients

This retrospective study was conducted in the Sint Maartenskliniek (SMK) and Radboud University Medical Centre (RUMC), which are both tertiary referral centres for orthopaedic care in The Netherlands. DAIRs performed from January 2012 to August 2021 and within 90 days after aseptic revision arthroplasty (index revision) of the hip (total hip arthroplasty, THA) or knee (total knee arthroplasty, TKA) were identified by surgical procedure codes. Revision arthroplasty was defined as a surgical procedure in which any component of the prosthetic joint was added or changed. DAIR after index revision was performed if there was a clinical suspicion of PJI based on typical signs of inflammation or persistent wound drainage (5–10 days after index revision). Patients were included if PJI was diagnosed based on tissue cultures taken during DAIR procedures, according to the IDSA guideline.^7^ Patients were excluded if they received antimicrobial treatment between index revision and DAIR, other than a short course of surgical prophylaxis. The start of the infectious episode was recorded as the day on which the DAIR procedure was performed. Data were collected by chart review and included comorbidity diseases, laboratory results, culture results (including antimicrobial susceptibility), antimicrobial treatment, type and duration of surgery and outcome data at the latest follow-up according to the outcome definition. Approval of the hospital ethical review committee was obtained.

### DAIR procedure

When performing the DAIR, the previous scar was re-excised completely with a full arthrotomy. Four to six intraoperative periprosthetic tissue samples from the synovium and capsule were obtained routinely with separate clean instruments. Subsequently, debridement and exchange of mobile parts was performed. The joint and wound were thoroughly irrigated with 6 L of saline, containing povidone using the pulse lavage system. After this, the capsule and subcutaneous tissue was meticulously closed with resorbable sutures and the skin with non-resorbable sutures.

### Microbiology and antimicrobial treatment

Orthopaedic samples were inoculated onto both a solid and a broth culture medium. Aerobic and anaerobic agar plates were incubated for 10–14 days, and broth was subcultured if it became turbid. Determination of microorganisms was performed using the MALDI-TOF. Clinical breakpoints as determined by EUCAST were used for defining resistance.^8^ For the index revision, prolonged cefazolin prophylaxis (single preoperative dose 2000 mg followed by 1000 mg thrice daily, 5 days in total) was used in the SMK, whereas a single preoperative cefazolin dose (2000 mg) was used in the RUMC. After DAIR procedure for suspected PJI, immediate empirical antimicrobial treatment was started. Treatment regimens were discussed by a multidisciplinary team and were ultimately targeted to culture results of tissue samples taken during DAIR. If at least one causative microorganism in a polymicrobial PJI was resistant to the empirical antimicrobial regimen, then the empirical regimen was defined as an antimicrobial mismatch. Targeted treatment was defined as the antibiotic regimen to which all the causative microorganisms of PJI were susceptible. DAIR was repeated if there was a clinical deterioration or in case of persistent wound leakage after the first DAIR and was considered as part of treatment if the second DAIR was performed during antimicrobial treatment. Antimicrobial treatment was continued until 3 months after the latest surgical procedure that was necessary to control the PJI.

### Outcome

The primary outcome parameter of the study was the antimicrobial mismatch rate of the empirical treatment defined as the number of cases in which at least one of the causative microorganisms of PJI, according to the IDSA guideline, was resistant to the empirical antimicrobial treatment divided by the total number of PJI cases. Secondary outcome parameters were the aetiology of PJI, and factors associated with antimicrobial mismatch or failure. As the last patient could be included up to August 2021, the follow-up period of several patients was less than 1 year after DAIR. The outcome of PJI treatment was analysed for those cases in which follow-up details for at least 2 years were available.

Failure was defined as:

- Repeated revision arthroplasty, implant removal or amputation at any moment following DAIR;
- Persistent or recurrent PJI according to the IDSA guideline after completion of the antimicrobial treatment;
- The use of suppressive antimicrobial treatment;
- Mortality related to failure of the implant.

If a repeated revision, implant removal or amputation was indicated but not performed for any reason, the outcome of that case was registered as failure.

### Statistical analyses

Data were reported as means and SD or medians and IQR as appropriate. Chi-square statistics or Fisher’s exact test was used to analyse the difference between groups for categorical variables, and a Student’s *t*-test or Mann–Whitney *U*-test was used for continuous variables. Success rate was analysed using Kaplan–Meier survival curves, including log-rank testing. Multivariable binary regression analysis determining risk factors for antimicrobial mismatching and failure of PJI treatment was performed if univariable testing showed a *P* value <0.10. Statistical significance was defined as a two-tailed *P* value <0.05. Statistical analyses were performed using IBM SPSS statistics version 25.0.0.1. Kaplan–Meier curves were constructed using Strata/IC 13.1 (StataCorp LLC).

## Results

### Patients

A total of 164 DAIRs performed within 90 days after aseptic revision arthroplasty were identified (Figure S1, available as Supplementary data at *JAC-AMR* Online). Forty-five cases were excluded because: no PJI was diagnosed (*n* = 10), no empirical antimicrobial treatment was started (*n* = 2), no tissue cultures were taken during DAIR procedure (*n* = 1) and antimicrobial treatment other than prophylaxis was used between revision and DAIR procedure (*n* = 32). As a result, a total of 119 cases were available for analysis. Fifty-five percent (66/119) of the patients were male, and the mean age at time of DAIR was 67 years (SD: 10). The mean BMI of patients in SMK was higher than in RUMC: 31 (SD: 5) and 29 (SD: 5), respectively (OR: 1.97; 95% CI: 0.05–3.88; *P* = 0.044). In RUMC 95% (42/44) of the index revisions were hip revisions, which was significantly more than the 56% (42/75) for hip revisions performed in SMK (OR: 15.5; 95% CI: 3.72–73.21, *P* < 0.001). Other patient characteristics between patients from both hospitals were not significantly different. The median time between the index revision and DAIR was 16 days (IQR: 12–24). Baseline patient characteristics are listed in Table 1.

**Table 1.** Patient characteristics and surgical characteristics for all patients included

	Total *n* = 119	Non-mismatch *n* = 33	Antimicrobial mismatch *n* = 86	*P* value (OR; 95% CI)	Adjusted *P* value (OR; 95% CI)
Centre, *n* (%)				0.610	
One	44 (37)	11 (33)	33 (38)		
Two	75 (63)	22 (68)	53 (62)		
Age (years), mean (SD)	67 (10)	64 (12)	69 (9)	**0.041 (2.07; 0.18–8.37)**	0.202
Male gender, *n* (%)	66 (55)	22 (67)	44 (51)	0.128	
BMI (kg/m²) mean (SD)	30 (5)	30 (4.6)	30 (5.4)	0.52	
Comorbidity, *n* (%)					
Rheumatic arthritis	20 (17)	3 (9)	17 (20)	0.272	
Diabetes mellitus	12 (10)	2 (6)	10 (12)	0.507	
Immunosuppression	12 (10)	0	12 (14)	**0.035 (1.45; 1.27–1.64)**	0.998
Joint hip, *n* (%)	84 (71)	25 (76)	59 (69)	0.443	
Index revision, *n* (%)					
Partial revision	79 (66)	26 (79)	53 (62)		
Total revision	11 (9)	2 (6)	9 (11)		
Conversion	4 (3)	0	4 (5)		
Reimplantation	25 (21)	5 (15)	20 (23)		
Nr index, median (IQR)	2 (1–3)	2 (1–3)	2 (1–3)	0.938	
Polymicrobial, *n* (%)	51 (43)	5 (15)	46 (53)	**<0.001 (6.44; 2.27–18.25)**	**<0.001 (6.89; 2.38–19.53)**
Delay in targeted treatment (days), median (IQR)	2 (0–5)	0 (0–0)	4 (2–6)		

Nr index, consecutive number of arthroplasties of the index procedure. **Statistical significance was defined as a two-tailed *P*-value <0.05.**

### Microbiology

Microorganisms causing PJI are listed in Table S1. Gram-positive microorganisms were identified in 92% (109/119) of the cases. The most common cultured Gram-positive microorganisms were *Staphylococcus epidermidis* (*n* = 63), *Staphylococcus aureus* (*n* = 26) and *Corynebacterium* spp. (*n* = 22). Gram-negative bacilli (GNB) were identified in 31% (37/119) of the cases. The most common cultured GNB were *Pseudomonas* (*n* = 12), *Enterobacter* spp. (*n* = 9) and *Klebsiella* spp. or *Proteus* spp. (*n* = 6 each). Polymicrobial PJI was diagnosed in 43% (51/119) of the cases. Polymicrobial PJI was significantly more often seen in RUMC than in SMK, despite correcting for the significant difference in BMI and number of hip revisions performed in patients of both hospitals (OR: 6.84; 95% CI: 2.67–17.51; *P* = <0.001). The most frequently identified causative microorganisms in polymicrobial PJI were staphylococci (*n* = 42), GNB (*n* = 28), corynebacteria (*n* = 21) and enterococci (*n* = 18). Only one case of PJI was caused by *Candida albicans* in a polymicrobial PJI case (Table S2).

### Empirical antimicrobial treatment and antimicrobial mismatch

**Figure 1.**
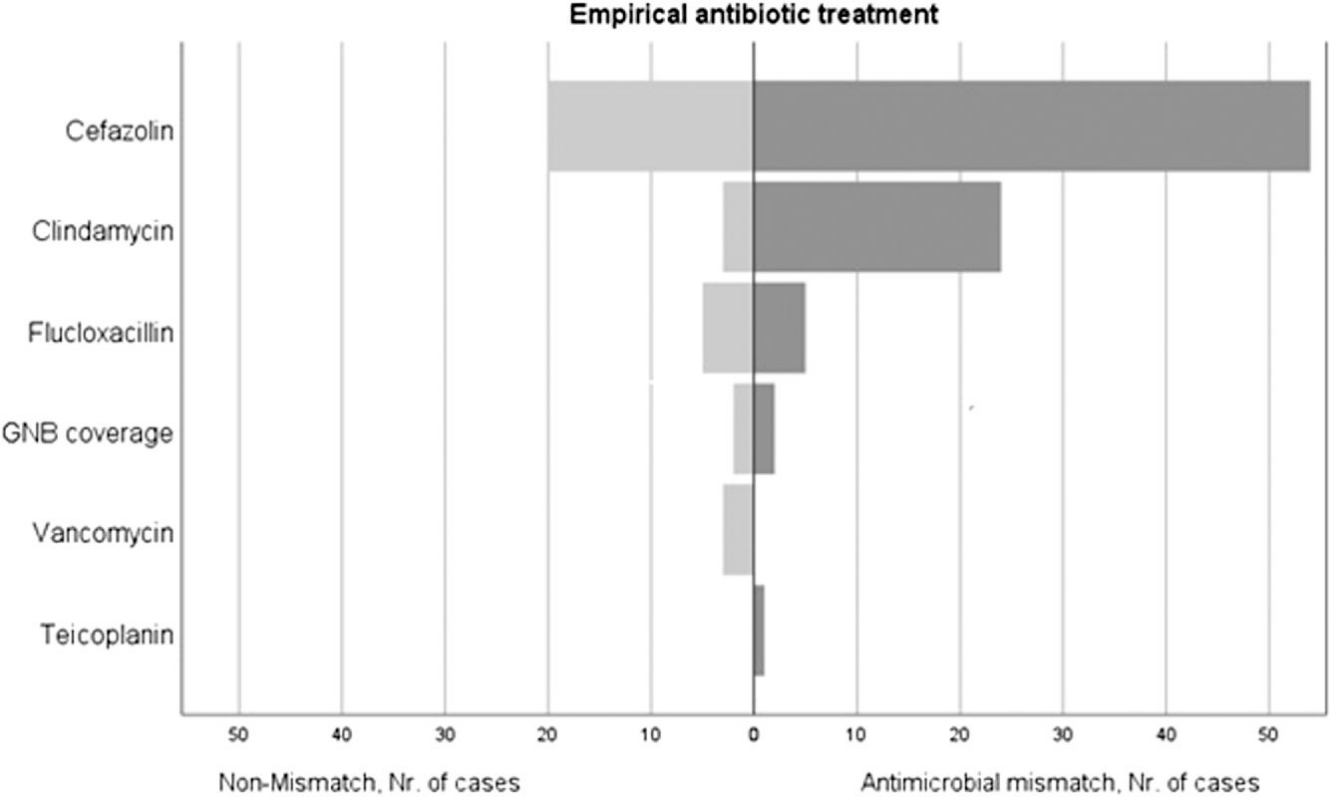
Number of antimicrobial mismatches of empirical treatment. Nr, number.

In all PJI cases, empirical antimicrobial treatment was started directly following DAIR. Overall, cefazolin (*n* = 74) was the most common empirical antimicrobial regimen used and mainly prescribed in SMK. Clindamycin (*n* = 26) was mainly prescribed in RUMC. Empirical antimicrobial treatment covering GNB was used in 3% (4/119) of the cases. In 72% (86/119) of the cases there was an antimicrobial mismatch between the empirical treatment and causative microorganisms of PJI (95% CI: 0.64–0.80) (Figure 1). The majority of the antimicrobial mismatches were caused by MDR *Staphylococcus* spp. (77%, 66/86), GNB (42%, 36/86), *Corynebacterium* spp. (24%, 21/86) and *Enterococcus* spp. (21%, 18/86). The antibiotic susceptibility rates of microorganisms are shown in Table 2. No VRE, MRSA or MDR GNB were found. No GNB were fluoroquinolones resistant. Thirty percent of the GNB (11/37) were co-trimoxazole resistant, which was due to *Pseudomonas aeruginosa* in all but one of these cases.

**Table 2.** Antimicrobial susceptibility: resistance rate of causative microorganisms of periprosthetic joint infections defined as the number of cases in which the microorganism was resistant devided by the total number of cases in which the microorganism was identified as causative

	Total *n* = 119	Polymicrobial	CFZ	VAN	RIF	TEC	CLI	CIP	COTR
*Staphylococcus* spp., *n* (%)	93 (78)	42/93	58/93	0/93	18/93	15/93	59/93	54/93	31/93
*S. epidermidis*	63 (53)	31/63	54/63	0/63	16/63	15/63	50/63	46/63	29/63
*S. lugdunensis/S. aureus*	28 (24)	14/28	0/28	0/28	1/28	0/28	8/28	6/28	2/28
*Enterococcus* spp*., n (%)*	19 (16)	18/19		0/19					
Gram-negative bacilli, *n* (%)	37 (31)	28/37						0/37	11/37^a^
*Corynebacterium* spp*., n* (%)	22 (18)	21/22		0/22					

CFZ, cefazolin; CIP, ciprofloxacin; CLI, clindamycin; COTR, co-trimoxazole; RIF, rifampicin; TEC, teicoplanin; VAN, vancomycin.

a 10/11 *Pseudomonas aeruginosa*, 1/11 *Proteus* spp. Antimicrobial susceptibility according to the clinical breakpoint determined by the EUCAST.

In the antimicrobial mismatch group, the median delay between DAIR and targeted treatment was 4 days (IQR: 2–6). More polymicrobial PJIs were seen in the antimicrobial mismatch group compared with the non-mismatch group: 53% (46/86) versus 15% (5/33), respectively (OR: 6.44; 95% CI: 2.27–18.25; *P* < 0.001). In multivariable binary regression analysis, polymicrobial infection persisted to be significantly associated with antimicrobial mismatch (OR: 6.89; 95% CI: 2.38–19.53; *P* < 0.001). If vancomycin had been used as empirical antimicrobial treatment in all cases, the antimicrobial mismatch rate would have been reduced to 32% (38/119; 95% CI: 0.24–0.40). If vancomycin and ciprofloxacin combination treatment had been used in all cases, only one antimicrobial mismatch would have occurred, due to the *C. albicans* infection (<1%).

### Outcome

**Figure 2.**
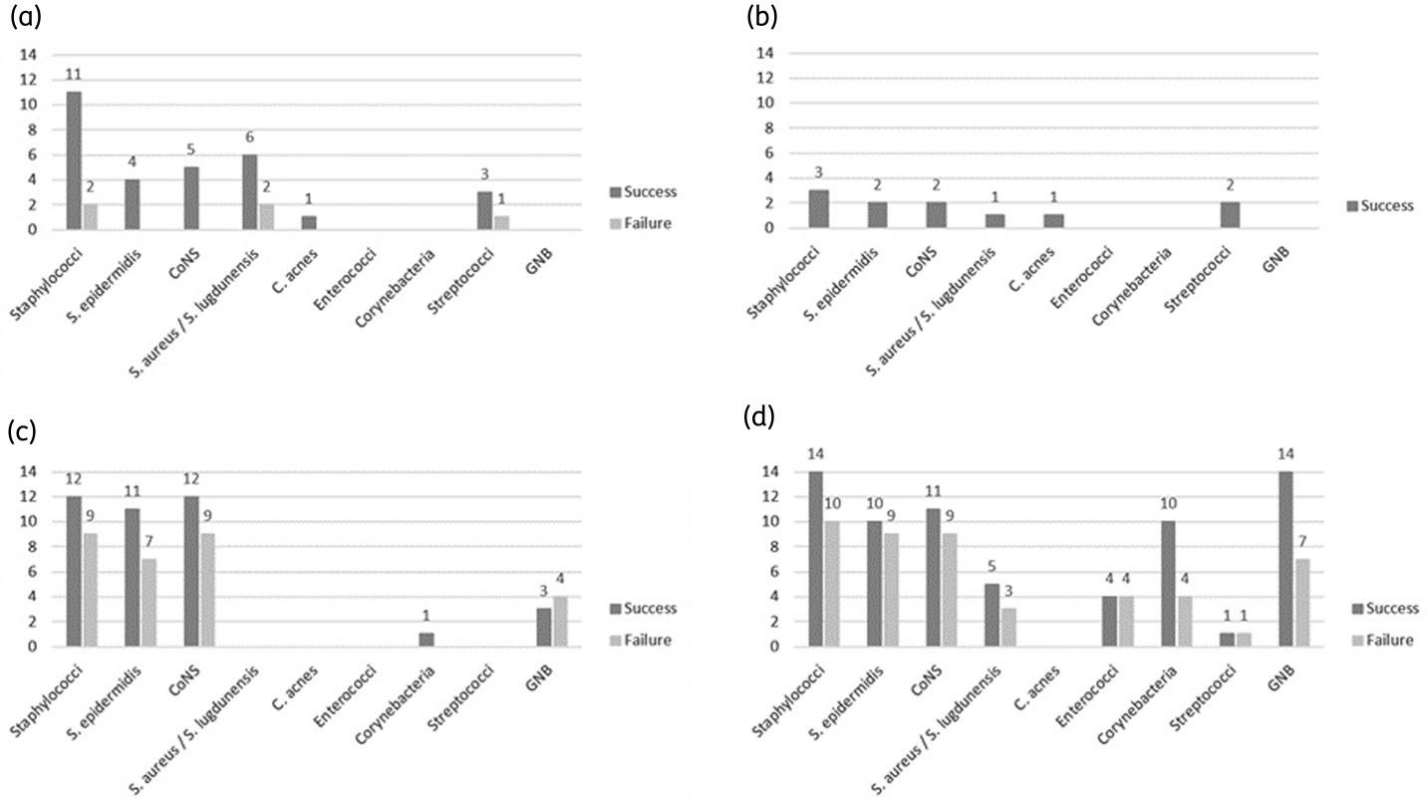
(a) Monomicrobial periprosthetic joint infections, no antimicrobial mismatch of empirical treatment. (b) Polymicrobial periprosthetic joint infections, no antimicrobial mismatch of empirical treatment. (c) Monomicrobial periprosthetic joint infections, with antimicrobial mismatch of empirical treatment. (d) Polymicrobial periprosthetic joint infections, with antimicrobial mismatch of empirical treatment. C. acnes, *Cutibacterium acnes*; CoNS, coagulase-negative Staphylococci; GNB, Gram-negative bacilli; S. epidermidis, *Staphylococcus epidermidis*; S. aureus, *Staphylococcus aureus*; S. lugdunenesis, *Staphylococcus lugdunensis.*

For 81 cases 2-year follow-up details after DAIR were available (Table 3). One person died because of sepsis, which might have been related to PJI. Antimicrobial mismatches were significantly associated with failure of 42% (25/60) compared with 14% (3/21) in non-mismatches (OR: 4.29; 95% CI: 1.14–16.13; *P *= 0.032) (Figure S2). In multivariable binary regression analysis correcting for age, male gender, polymicrobial infection and antimicrobial mismatch on the outcome of PJI treatment, antimicrobial mismatch persisted in being independently associated with failure (OR: 5.102; 95% CI: 1.22–21.43; *P = *0.026). Figure 2 shows the outcome of monomicrobial and polymicrobial PJI treatment according to the causative microorganisms. The success rate of PJI treatment in which GNB were (part of) the causative microorganisms was comparable to the success rate of that in which Gram-positive microorganisms were (part of) the causatives: 61% (17/28) versus 68% (36/53), respectively (*P* = 0.516).

**Table 3.** Success rate of periprosthetic joint infection treatment after 2-year follow-up

	Total *n* = 81	Success *n* = 53	Failure *n* = 28	*P* value (OR; 95% CI)	Adjusted *P* value
Centre, *n* (%)				0.635	
One	29 (36)	18 (34)	11 (39)		
Two	52 (64)	35 (66)	17 (61)		
Male gender, *n* (%)	47 (58)	32 (60)	15 (54)	0.555	0.981
Age (years), mean (SD)	66 (11)	66 (11)	66 (10)	0.920	0.701
BMI (kg/m²), mean (SD)	30 (6)	30 (6)	31 (6)	0.447	
Comorbidity, *n* (%)					
Rheumatoid arthritis	10 (12)	6 (11)	4 (14)	0.731	
Diabetes mellitus	9 (11)	8 (15)	1 (4)	0.153	
Immunosuppressive	7 (9)	4 (8)	3 (11)	0.688	
Joint, hip, *n* (%)	54 (67)	36 (68)	18 (64)	0.741	
Nr index, median (IQR)	2 (1–3)	2 (1–4)	1 (1–3)	0.747	
Interval index to DAIR (days), median (IQR)	16 (12–24)	16 (12–21)	16 (11–31)	0.713	
Antimicrobial mismatch, *n* (%)	60 (74)	35 (66)	25 (89)	**0.032 (4.29; 1.14–16.13)**	**0.026 (5.102; 1.215–21.429)**
Delay in targeted treatment (days), median (IQR)	3 (0–6)	2 (0–6)	3 (1–6)	0.106	
Polymicrobial PJI, *n* (%)	34 (42)	22 (42)	12 (43)	0.907	0.557
*Staphylococcus* spp., *n* (%)	61 (75)	40 (75)	21 (75)	0.963	
*S. epidermidis*	43	27	16	0.595	
*S. aureus/S. lugdunensis*	17	12	5	0.615	
*Cutibacterium acnes*, *n* (%)	2 (2)	2 (4)	0	0.542	
*Corynebacterium* spp*., n* (%)	15 (19)	11 (21)	4 (14)	0.560	
*Streptococcus* spp*., n* (%)	8 (10)	6 (11)	2 (7)	0.708	
*Enterococcus* spp*., n* (%)	8 (10)	4 (8)	4 (14)	0.438	
Gram-negative bacilli, *n* (%)	28 (35)	17 (32)	11 (39)	0.516	

Nr index, consecutive number of procedures on affected joint. **Statistical significance was defined as a two-tailed *P*-value <0.05.**

## Discussion

We observed a high number of antimicrobial mismatches between empirical treatment and the susceptibility of microorganisms causing PJI after aseptic revision arthroplasty treated with DAIR. Polymicrobial PJI was an independent risk factor for antimicrobial mismatch of the empirical treatment. Antimicrobial mismatch was significantly associated with failure of PJI treatment after 2-year follow-up. This study gives a unique insight into the aetiology, risk factors for and impact of antimicrobial mismatches in PJI after aseptic revisions treated with DAIR.

Staphylococci were the predominant microorganisms causing early PJI after aseptic revision arthroplasty in our study, which is also seen in PJI after primary arthroplasty.^4,9^ In the Netherlands, the MRSA rate is as low as <1% in the general population, and therefore early PJI after arthroplasty is rarely caused by MRSA. The antimicrobial mismatch rate between the empirical treatment and causative microorganism was as high as 72% in our cohort. This was mainly due to β-lactam-resistant *S*. *epidermidis* (MDR-SE), while cefazolin was predominantly used as empirical antimicrobial treatment. Although resistance patterns differ locally, the emerging resistance rate of *S. epidermidis* to β-lactam antibiotics in PJI is of concern.^10^ In the study of Hu *et al*.^11^ an increase in MDR-SE PJI from 39% to 62% was seen between 2006 and 2015. Triffault-Fillit *et al*.^12^ reported an MDR rate of coagulase-negative Staphylococci (CoNS) causing PJI of 59%. The proportion of revision arthroplasty in these studies was not mentioned. It is therefore not known if the incidence of MDR-SE differs between PJI after primary and revision arthroplasty. Besides the resistance rate of *S. epidermidis* to β-lactam antibiotics, we also found a high resistance rate of staphylococci to other frequently used antibiotic agents for PJI, like quinolones, rifampicin, co-trimoxazole, teicoplanin and clindamycin. In our cohort, clindamycin was also frequently used as empirical antimicrobial treatment. Unfortunately, we could not correct for antibiotic use prior to the index revision as a risk factor for antimicrobial resistance because we did not collect these data.

Enterococci (16%), corynebacteria (18%) and GNB (31%) were more often seen as causatives of PJI in our cohort than is observed in PJI after primary arthroplasty.^4,13,14^ Although some researchers question the relevance of *Corynebacterium* spp. as causative microorganisms in PJI, *Corynebacterium* spp. have increasingly been recognized as causative bacteria.^15^ Similar to previous reports, we found that the above mentioned microorganisms were mainly seen as part of polymicrobial PJI.^5,13,16^ Polymicrobial PJI was significantly associated with antimicrobial mismatch of the empirical treatment in our cohort. For unknown reasons, polymicrobial PJI was significantly more often seen in RUMC, despite correcting for the difference in patients and surgical characteristics between the hospitals. The extended antibiotic prophylaxis that has been used in SMK could be an explanation for the difference in polymicrobial PJIs. The prolonged administration of cefazolin could have resulted in colonization of the skin with β-lactam-resistant microorganisms that are more often seen in polymicrobial infection.^17^ The high incidence of β-lactam-resistant staphylococci and polymicrobial PJI as risk factors for antimicrobiral mismatches makes vancomycin a better choice for empirical antimicrobial treatment than cefazolin or clindamycin, even in the context of low prevelance of MRSA. Vancomycin, however, has been thought to be inferior to β-lactam antibiotics to treat PJI caused by staphylococci, in particular *S. aureus,* and has a risk of serious side effects like renal dysfunction.^18–20^ In our study, the outcome results of PJI caused by methicillin-susceptible *S. aureus, Streptococcus* spp. and *Cutibacterium* spp. were very good (MSSA). It is not known if the outcome results of PJI caused by staphylococci would be less favourable if vancomycin had been used as empirical treatment.

In our cohort, 31% of the PJIs were caused by GNB, which is a higher rate of GNB PJI than observed in PJI after primary arthroplasty.^13,21^ As SMK and RUMC are both tertial referral centres, more complex cases can be admitted there, which contribute to the risk of more complex PJIs such as polymicrobial and GNB PJI. There is a concern that the prognosis of GNB PJI is less favourable than the prognosis of PJI caused by Gram-positive bacteria, although recent studies show contradictory results.^22–24^ In none of the studies in which the outcome of GNB PJI was described, the mismatch in empirical treatment or delay in targeted treatment was included in the analysis. In our study, the outcome of GNB PJI was not significantly different from the outcome of PJI caused by Gram-positive microorganisms; however, the success rate of Gram-positive PJI was reduced by a high number of mismatches. Because of the low number of GNB PJI cases without antimicrobial mismatch, we were not able to calculate the impact of antimicrobial mismatch on the outcome of GNB PJI. It is not known if the antimicrobial mismatch in GNB PJI contributes to a reduced success rate of GNB PJI, and if broadening the empirical treatment would improve the success rate.^25,26^. Further research is necessary to find out if the assumed less favourable prognosis of GNB PJI is caused by bacterial factors or by antimicrobial mismatch in empirical treatment causing a delay in targeted treatment. Because broadening the empirical antimicrobial treatment has an increased risk of toxicity and risk for selection and induction of antimicrobial resistance, we suggest focusing on speeding up the identification of the causative microorganisms. If rapid identification of causatives (i.e. next-generation sequencing) is possible, GNB coverage can be preserved for those patients in which early culture results indicate a GNB, awaiting susceptibility information.^21,27–29^ When a broad-spectrum antibiotic regimen is indicated, ceftaroline can be a good option for PJI treatment.^30^ This novel agent is a fifth-generation cephalosporin that is highly effective against MRSA and MDR-SE and also Enterobacterales.^31^*In vitro* studies showed good penetration of ceftaroline into the synovial fluid and bone tissue.^32^ Prospective clinical studies are needed to investigate its exact role in PJI treatment.

Our study has several limitations. First, this is a retrospective study only considering PJI treated with DAIR after aseptic revision arthroplasties. This means that extrapolation of the results to other types of arthroplasties should be done with caution. Second, SMK has used extended prophylaxis between index revision and DAIR, and both centres are tertial reference centres, which might influence the aetiology of PJI. The difference in empirical antimicrobial treatment that has been used between the hospitals may account for confounding bias. Third, because of the small numbers in this cohort, we risk overinterpretation. Despite these limitations, we believe our results are unique and important to re-evaluate the way we interpreted treatment options for PJI.

In conclusion, a high antimicrobial mismatch rate between the empirical treatment and the susceptibility of mainly the Gram-positive microorganisms of early PJI after aseptic revision arthroplasty was observed, especially in polymicrobial PJI. Vancomycin is a better choice for empirical Gram-positive coverage. More research is necessary to identify the effect of GNB coverage to the empirical treatment on the outcome results of GNB PJI. We suggest focusing on strategies speeding up microbiological testing where possible, to be able to preserve GNB coverage only for selected cases. Our study demonstrates that local review of the microbiological data is important to reflect on the local treatment protocols. We challenge researchers in future studies not only to focus on the susceptibility patterns of pathogens, but also to relate the antimicrobial mismatch and delay in targeted treatment to the outcome results. Antimicrobial mismatch and the delay in targeted treatment should be integrated in the approach to optimize clinical outcomes while minimizing unintended consequences of antimicrobial use (antimicrobial stewardship).

## Supplementary Material

dlac124_Supplementary_Data

## Supplementary data

Figures S1 and S2 and Tables S1 and S2 are available as Supplementary data at *JAC-AMR* Online.
