# Supplementary material for: Assessment of antimicrobial mismatches in empirical treatment in early PJI after aseptic revision arthroplasty
Source: JAC Antimicrob Resist. 2022 Dec 7;4(6):dlac124. doi: 10.1093/jacamr/dlac124 (PMC9728518; doi:10.1093/jacamr/dlac124)
Supplement: dlac124_Supplementary_Data [file dlac124_supplementary_data.docx]

**Figure S1:** Inclusion of DAIR procedures performed within 90 days after index revision arthroplasty of the hip or knee.
DAIR, debridement antimicrobial treatment and implant retention; PJI, periprosthetic joint infection


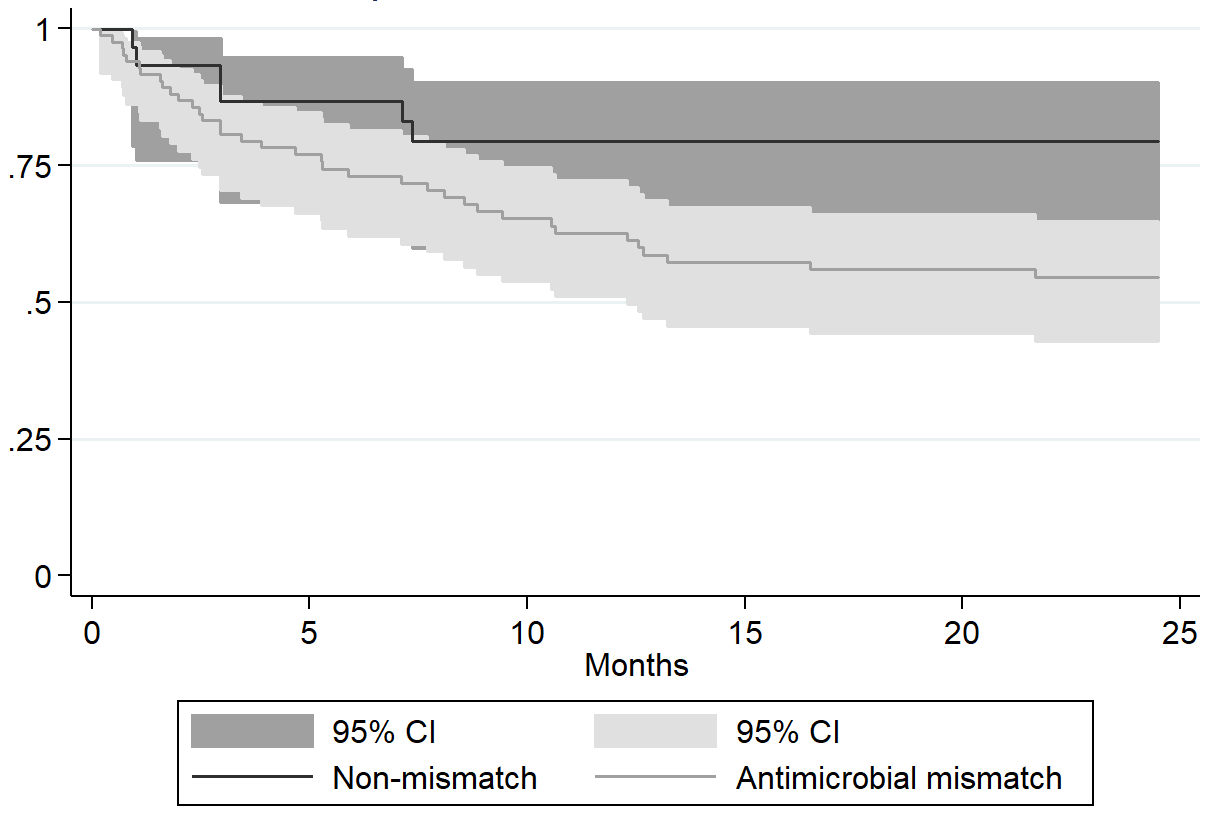


**Figure S2:** Success rate of periprosthetic joint infection treatment after 2 years follow-up, according to matching empirical antimicrobial treatment. Log-rank p=0.037
CI, confidence interval

**Table S1:** Aetiology of periprosthetic joint infection for all patients included

| Microorganism | Total n=119 | Non-mismatch  n=33 | Antimicrobial mismatch  n=86 | *P*-value  (OR; 95% CI) | Adjusted p-value  (OR: 95% CI) |
| --- | --- | --- | --- | --- | --- |
| *Staphylococcus spp*., n (%) | 93 (78) | 27 (82) | 66 (77) | 0.549 |  |
| *S. epidermidis* | 63 | 10 | 53 | **0.002 (3.69; 1.56-8.73)** |  |
| CoNS other | 9 | 2 | 8 | 0.724 |  |
| *S. aureus / S. lugdunensis* | 29 | 16 | 13 | **<0.001 (0.19; 0.08-0.47)** |  |
| *Cutibacterium acnes,* n (%) | 5 (4) | 3 (9) | 2 (2) | 0.130 |  |
| *Enterococcus spp.,* n (%) | 19 (16) | 1 (3) | 18 (21) | **0.023 (8.47; 1.08-66.26)** |  |
| *E. faecalis* | 15 | 1 | 14 |  |  |
| *E. faecium* | 5 | 0 | 5 |  |  |
| *Corynebacterium spp,* n (%) | 22 (18) | 1 (3) | 21 (24) | **0.007 (10.34; 1.33-80.33)** |  |
| *Streptococcus spp.* n (%) | 11 (9) | 7 (21) | 4 (5) | **0.010 (0.18; 0.05-0.67)** |  |
| Gram-negative bacilli, n (%) | 37 (31) | 1 (3) | 36 (42) | **<0.001 (23.04; 3.01-176.47)** |  |
| *Pseudomonas spp.* | 12 | 0 | 12 |  |  |
| *Enterobacter spp.* | 9 | 0 | 9 |  |  |
| *Klebsiella spp.* | 6 | 0 | 6 |  |  |
| *Proteus spp.* | 6 | 1 | 6 |  |  |
| *Acinetobacter spp.* | 2 | 0 | 2 |  |  |
| *Escherichia coli* | 2 | 0 | 2 |  |  |
| *Morganella spp.* | 2 | 0 | 2 |  |  |
| *Haemophilus spp.* | 2 | 0 | 2 |  |  |
| *Pasteurella spp.* | 1 | 0 | 1 |  |  |
| *Citrobacter spp.* | 1 | 1 | 0 |  |  |
| *Serratia spp.* | 2 | 0 | 2 |  |  |
| *Candida albicans* | 1 (<1) | 0 | 1 |  |  |

95% CI: 95% confidence interval; CoNS, coagulase negative Staphylococci; IQR, interquartile range; n, number; OR, odds ratio; SD, Standard deviation; spp., species

**Table S2:** Characteristics of monomicrobial and polymicrobial periprosthetic joint infections

|  |  | Monomicrobial  n=68 | Polymicrobial  n=51 | *P*-value  (OR; 95% CI) |
| --- | --- | --- | --- | --- |
| Centre, n (%) |  |  |  | **<0.001 (0.18; 0.08-0.41)** |
| One |  | 14 (21) | 30 (59) |  |
| Two |  | 54 (79) | 21 (41) |  |
| Age year, mean (SD) |  | 66 (11) | 70 (9) | 0.051 |
| Male gender, n (%) |  | 35 (51) | 31 (61) | 0.312 |
| BMI kg/m², mean (SD) |  | 30 (5) | 30 (5) | 0.618 |
| Comorbidity, n (%) |  |  |  |  |
| Rheumatic arthritis |  | 13 (19) | 7 (14) | 0.436 |
| Diabetes mellitus |  | 8 (12) | 4 (8) | 0.552 |
| Immunosuppression |  | 8 (12) | 4 (8) | 0.552 |
| Joint hip, n (%) |  | 44 (65) | 40 (78) | 0.104 |
| Nr. index, median (IQR) |  | 2 (1-4) | 1 (1-3) | 0.910 |
| *Staphylococcus spp.,* n (%) |  | 51 (75) | 42 (82) | 0.337 |
| *S. epidermidis* |  | 32 | 31 | 0.138 |
| *S. aureus / S. lugdunensis* |  | 15 | 14 | 0.498 |
| *Cutibacterium acnes,* n (%) |  | 2 (3) | 3 (6) | 0.650 |
| *Enterococcus spp.*, n (%) |  | 1 (1) | 18 (35) |  |
| *Corynebacterium spp.*, n (%) |  | 1 (1) | 21 (42) | **<0.001 (46.90; 6.03-364.96)** |
| *Streptococcus spp.,* n (%) |  | 4 (6) | 7 (14) | 0.202 |
| Gram-negative bacilli, n (%) |  | 9 (13) | 28 (55) | **<0.001 (7.98; 3.27-19.48)** |
| Antimicrobial mismatch, n (%) |  | 40 (59) | 46 (90) | **<0.001 (6.44; 2.27-18.25)** |

95% CI: 95% confidence interval; BMI, body mass index; IQR, interquartile range; n, number; Nr index: consecutive number of procedures on affective joint; OR, odds ratio; SD, Standard deviation; spp., species
